# Low-Carbohydrate Diets for Gestational Diabetes

**DOI:** 10.3390/nu11081737

**Published:** 2019-07-27

**Authors:** Sarah S. Farabi, Teri L. Hernandez

**Affiliations:** 1 Goldfarb School of Nursing, Office of Nursing Research, Barnes-Jewish College, St. Louis, MO 63110, USA; 2 Department of Medicine, Division of Nutritional Science, Washington University in St. Louis, St. Louis, MO 63130, USA; 3 Department of Medicine, Division of Endocrinology, Metabolism, and Diabetes, Anschutz Medical Campus, University of Colorado, Aurora, CO 80045, USA; 4 College of Nursing, Anschutz Medical Campus, University of Colorado, Aurora, CO 80045, USA; 5 Department of Research, Innovation, and Professional Practice, Children’s Hospital Colorado, Anschutz Medical Campus, University of Colorado, Aurora, CO 80045, USA

**Keywords:** pregnancy, gestational diabetes, low-CHO, obesity, nutrition, diet

## Abstract

Nutrition therapy provides the foundation for treatment of gestational diabetes (GDM), and has historically been based on restricting carbohydrate (CHO) intake. In this paper, randomized controlled trials (RCTs) are reviewed to assess the effects of both low- and higher CHO nutrition approaches in GDM. The prevailing pattern across the evidence underscores that although CHO restriction improves glycemia at least in the short-term, similar outcomes could be achievable using less restrictive approaches that may not exacerbate IR. The quality of existing studies is limited, in part due to dietary non-adherence and confounding effects of treatment with insulin or oral medication. Recent evidence suggests that modified nutritional manipulation in GDM from usual intake, including but not limited to CHO restriction, improves maternal glucose and lowers infant birthweight. This creates a platform for future studies to further clarify the impact of multiple nutritional patterns in GDM on both maternal and infant outcomes.

## 1. Introduction

Gestational diabetes is one of the most intensely debated topics in obstetric history. Although its recognition emerged in the post WWII era [1], it was only within the last 10–15 years that two pivotal RCTs demonstrated improved perinatal outcomes from diagnosis and treatment [2,3]. Evidence from these trials supported the inclusion of GDM screening in standard-of-care protocols for pregnancy world-wide, ending decades of controversy. In 2010, however, new diagnostic criteria for GDM [4] were proposed based on data from the landmark Hyperglycemia and Adverse Pregnancy Outcomes (HAPO) study [5]. Since the HAPO study, the field of diabetes in pregnancy again finds GDM as the focus of debate; international consensus on diagnostic criteria has not been reached nearly a decade later [6], and first-line medical [pharmaceutical] therapy has recently been called into question [7,8]. Because GDM has both short-term and lasting effects on maternal and offspring metabolic health, the stakes are high to determine and reach consensus on consistent, effective strategies that improve lifelong health in this important population.

Fortunately, across diagnostic criteria, the universal first-line treatment for GDM is nutrition therapy [9,10]. Based on decades of clinical experience in which diet was manipulated to treat diabetes outside of pregnancy [11,12], the suggested primary strategy for treatment of GDM was the restriction of dietary carbohydrate (CHO) [13]. Although there is little evidence that supports this approach in GDM [9,14], low-CHO diets remain the conventional treatment in some parts of the world [15]. Rigid CHO restriction carries a risk for replacement of CHO energy with that from fat due to increased availability of processed foods and a tendency for over-nutrition [16]. This is concerning as our group [17] and others [18] have demonstrated a link between increasing maternal lipids and patterns of fetal overgrowth. More than a decade ago [19], international consensus on CHO restriction in GDM was abated due to concerns that higher maternal fat consumption (total and saturated) could exacerbate maternal insulin resistance (IR), potentially resulting in even higher glucose levels [19], and could also promote increased availability of all maternal nutrients (CHOs, fats, and amino acids) to the fetal-placental unit. Although limited, there are a number of formative studies using both restrictive and less-restrictive approaches to CHO consumption that have informed our contemporary understanding of nutrition therapy in GDM. In this paper, these RCTs will be reviewed to highlight the known effects of both approaches as first-line therapy for GDM.

## 2. Perspective: Obesity in Pregnancy Drives Gestational Diabetes Prevalence

Obesity prevalence in young women worldwide continues to change the landscape of pregnancy [9]. Obesity affects 25–40% [20,21] of young women worldwide, and fetal overgrowth is largely accounted for by pregnancies complicated by obesity alone and not GDM [22]. In a concerning pattern seen across populations, more infants begin life with higher birthweight and/or adiposity. Further, many children have accelerated growth early in life and have heightened adiposity or develop metabolic syndrome. Superimposed on this pattern may be years of poor or excessive nutrition, reduced sleep quality and sedentarity [23,24]. Before pregnancy begins, these patterns interact to produce a phenotype which includes mild hyperlipidemia and hyperglycemia due to IR and obesity, upon which the metabolic adaptations of pregnancy are additive [25]. Gestational diabetes now impacts ≥20% of pregnancies in some parts of the world [26] and approaches a 40% prevalence within obese women in Europe [27]. Although the GDM phenotype is highly heterogeneous [28], half of its prevalence can be explained by overweight and obesity [29,30]. More concerning is that prospective RCTs in which lifestyle changes, including nutrition, were initiated during early pregnancy and have been unsuccessful overall in preventing GDM in at-risk obese women [31,32]. Postpartum follow-up of these mothers tends to be poor [33]. It was recently identified in the HAPO cohort that 11 years after GDM, the higher incidence of childhood overweight/obesity, increased adiposity, and larger waist circumference was explained by pre-pregnancy BMI [34]. This highlights the need for effective interventions targeting obesity and lifestyle that reduce the metabolic burden earlier in life, well before motherhood, and underscores that such interventions may benefit both the mother and future offspring.

## 3. History and Rationale for Nutritional Therapy in Gestational Diabetes

The rationale behind CHO restriction as nutrition therapy for GDM is rooted in the pre-insulin era [35]. During this time, leaders in the field including Joslin and Allen observed that extreme CHO restriction to 8–10% of total caloric intake (>70% fat) was a powerful intervention that could prolong life in those with Type 1 diabetes [12]. Also, during this era, the incidence of offspring overgrowth in maternal diabetes, which often resulted in tragic stillbirth, was documented and over time associated with maternal glucose [1,36]. Thus, it has long been recognized that fetal overgrowth and macrosomia are critical concerns in pregnancies complicated by diabetes, and are linked to maternal patterns of glycemia [1].

During the WWII era, White [1] and Pedersen [37] (reviewed in Ref. [11]) developed strict protocols for glucose control in (Type 1 diabetes) pregnancy that resulted in improved maternal outcomes and a marked reduction in infant mortality. In the following decades, as the GDM phenotype emerged [38], Freinkel and others characterized in-utero conditions as dominantly influenced by maternal nutrition [36]. The culmination of evidence from these formative years and seminal studies supported a strong association between maternal fasting and postprandial glycemia with fetal overgrowth [13] that has been reinforced by contemporary evidence [5]. In 1990 [13], Jovanovic proposed CHO restriction as first-line therapy for GDM—a suggestion that made sense based on the decades of experience (and success) in diabetes outside of pregnancy. By then it had been described that metabolism during later pregnancy, when GDM is diagnosed, is exemplified by IR that exaggerates the availability of maternal fuels to support increasing fetal growth [39], featuring increased postprandial glucose [36], a two- to three-fold increase in insulin secretion [30,40], and increased TG and FFA [30,40,41]. Freinkel and Metzger [36] coined the term *facilitated anabolism* to describe heightened postprandial glycemia in pregnancy. In *facilitated anabolism*, the maternal-fetal glucose gradient is increased by rising glucose postprandially which allows for glucose to be transported from the mother to the fetus. Thus, it was recognized that maternal postprandial glucose provides direct nourishment to the fetus [42]. Subsequently, in GDM, it was rationalized that the restriction of CHO to 30–40% could mitigate fetal overgrowth by reducing postprandial hyperglycemia [13]. An average of 175 g of CHO per day is recommended to all pregnant women for placental requirements, to prevent maternal ketosis, and also to account for additional glucose to support fetal brain development (33 g/day) [43,44]. Supported by only two small studies [45,46], one of which was randomized, CHO restriction in GDM remained the mainstay of therapy until the 5^th^ International Conference on GDM in 2005, where concerns were raised about higher maternal fat intake and fetal lipid exposure [19]

In hindsight, studies of nutrition in GDM in the last 2–3 decades have focused on demonstrating superiority in *type of diet* in relation to maternal glucose, rather than on the fundamental question of *any diet* (any manipulation from usual behavior), which actually mitigates maternal glucose and fetal overgrowth in GDM [35]. This was only recently supported by a meta-analysis [47]. Across 18 RCTs including 1151 women with GDM, Yamamoto and colleagues [47] demonstrated that nutritional manipulation intended to improve the dietary pattern from usual intake, including but not limited to CHO restriction, resulted in a reduction in fasting glucose (by 4 mg/dL), postprandial glucose (by 8 mg/dL), and birth weight (by 171 g; 9 studies). Overall, study heterogeneity was high and the quality of evidence was low, which is an assessment supported by others [2,48]. Unfortunately, a primary barrier to treatment adherence in GDM has now been identified as a restrictive approach to nutrition [16]. This has led to concerns regarding unintended consequences of restrictive/unbalanced nutrition [9,14,16,49] and supports that nutrition therapy tailored to the needs of the mother, regardless of type, may lead to better outcomes.

## 4. Evidence Review: Effects of RCTs of Carbohydrate Intake for Nutrition Therapy in GDM

### 4.1. Maternal Glucose Metabolism

Glucose targets for treatment of GDM are: fasting glucose of ≤90–95 mg/dL, 1 h postprandial glucose ≤140 mg/dL or 2-h postprandial glucose ≤120 mg/dL [4,11]. Achieving fasting glucose ≤95 mg/dL within 2 wks of nutrition therapy is associated with a reduced risk of pharmacological agents [50,51]. Two studies of low- vs. higher CHO intake (35–40% vs. 60–70% CHO) demonstrated that both the lower and higher CHO intake strategies resulted in the achievement of postprandial glucose targets in a short time period (3–4 days, n = 4 women) [52] and (n = 16) [53]). Both studies were controlled randomized crossover studies in which all of the food was provided. Our group [53] showed, using continuous glucose monitors (CGM), that the 24-h glucose area-under-the-curve (AUC) on the 40% CHO diet was 6% lower compared with the 60% CHO diet. However, conventional glycemic targets were achieved with both diets. A lower postprandial glucose after 2 wks on this diet (compared with baseline) was also shown among women in Poland randomized to a 60% total CHO diet [54]. Although only within-group comparisons were made, it was also shown that in the low-CHO group (45% CHO), postprandial glucose was lower compared to baseline. Randomization to a dietary approach to stop hypertension (DASH, low sodium) diet (~65% CHO) resulted in lower fasting glucose and improvement in an oral glucose tolerance test [55] response when compared to a diet that was ~55% CHO among women in Iran [56]. It should be noted that neither nutrition strategy was technically lower in CHO by definition (≤40%).

In the randomized crossover study by our group [53] (all meals provided [57]), we examined dietary effects after 6–7 wks and up to delivery on the glucose response. The fasting glucose of women randomized to the diet with higher complex CHO (60% of total calories similar in CHO, sugars and fiber similar to DASH) was decreased while the fasting glucose of women randomized to the lower CHO (40% of total calories) diet demonstrated *increased* fasting glucose. The lower CHO arm had a higher fat content (total and saturated) while protein was similar (15–20%) between the two arms in both of these studies. Increased FFA from the higher total fat content could have worsened IR and been the cause of the increase in fasting glucose in the lower CHO group [58]. Overall, findings from these studies highlight that the achievement of good glycemic control is possible with both restrictive and less CHO-restrictive nutrition therapy.

### 4.2. Lipids, Inflammation, and Insulin Resistance

Lipids, particularly FFA, and inflammation both contribute to whole-body IR. Over the short term (3–4 day) randomized crossover studies, the low-CHO diets (vs. higher CHO) increased fasting total cholesterol and FFA [52] and increased the postprandial response of FFA to a controlled meal [53], which could exacerbate IR. Conversely, HbA1c, systolic blood pressure and lipids were lowered after consumption of the DASH diet (65% CHO/18% fat) for 4 wks [55]. A reduction in oxidative stress, exhibited by increased antioxidant capacities and increased total glutathione, and improved IR were also seen [56]. Importantly, however, consumption of the DASH diet was not confirmed by urine sodium concentrations (a marker of treatment adherence). The intervention was not maintained throughout pregnancy [55,56] which could influence findings as IR significantly increases over the last trimester. In the study by our group [57], maternal adipose tissue was biopsied in late pregnancy (37 wks). We observed worsened isoproterenol-stimulated lipolysis (31% suppression), a marker of IR in adipose tissue, in women randomized to the lower (40%) CHO diet compared to suppression in the higher (60%) CHO arm (56% suppression). Gene expression in the adipose tissue of mothers on the lower CHO/higher fat diet also showed a pattern of higher inflammation [57]. These data suggest that exposure to a more CHO-restrictive diet worsens insulin action in adipose tissue after 6–7 wks. Overall, findings from these studies imply that the reduction of inflammation and lipids from a higher-quality complex CHO/lower total fat diet that is less restrictive may result in improved insulin action in GDM.

### 4.3. Insulin Treatment

It has been supported, in non-randomized studies, that the achievement of good glycemic control is possible using a combination of diet and insulin [1], and that there is a reduced need for insulin treatment with consumption of <42% CHO [59]. The outcome of need for insulin prescription was used to power two randomized control trials. Although not a study with a low-CHO arm [60], 59% of women randomized on a higher-GI diet (GI = 56; higher potential to increase postprandial glucose) required insulin while only 29% of women on the low-GI diet (GI = 48) did. Importantly, of the women who failed the higher GI diet, 50% of women were able to prevent insulin use by switching to the low-GI strategy. The single RCT of lower vs. higher CHO diet in GDM powered on the need for insulin was conducted in Spain [61]. In this study, the need for insulin was similar in both groups; 55% of women on the low-CHO (~40% CHO/40% fat) and higher CHO diet (~55% CHO/25% fat) required insulin. Unfortunately, total calories, protein and fat intakes were not reported. Moreover, intake of simple sugars was not decreased in either group over a 6–12 wk diet intervention. Randomization to the DASH for 4 wks (65% CHO/18% fat vs. 54% CHO/28% fat) resulted in a reduced need for insulin. Importantly, the diet was followed for only 4 wks and it is unknown if the women stayed on the diet for the remainder of pregnancy (8–10 wks) [62]. In summary, data from existing randomized studies do not support that lower CHO intake results in less need for insulin. A low-GI diet can reduce the need for insulin, and the DASH diet may show promise if the effect is confirmed.

### 4.4. Gestational Weight Gain (GWG)

Conventional dogma posits that increased CHO intake (50–60%) leads to postprandial hyperglycemia and excessive GWG [63], which further supports strict CHO reduction in GDM. Increased GWG would be a reasonable expectation with over-nutrition and high consumption of simple sugars, which increases plasma insulin concentrations and could promote lipid storage in adipose tissue [64]. However, increased caloric intake, regardless of macronutrient source, will be expected to lead to increased GWG. In a study in Spain [61], women randomized to the low-CHO arm (40% CHO/40% fat) gained 1.4 kg, compared to 2.3 kg in the 55% CHO group (25% fat). The same was true in the Australian GI study [65]: Of the women randomized to the higher GI group, 42% had excessive GWG [66], compared to only 25% of women in the low-GI group. Importantly, in both studies, weight gained during the interventional period is not certain as the authors only reported total GWG over the pregnancy. In the GI study from Australia, a high rate of insulin treatment in both GI groups further confounds the increased GWG for two reasons: first, weight gain is independently correlated with insulin therapy; and second, with insulin treatment comes a higher risk for hypoglycemia that could necessitate higher CHO intake for avoidance [9]. Three RCTs reported ~1–2 kg GWG during the dietary intervention period [55,56,57,62], on either a lower or higher CHO eucaloric diet, supporting the fact that when calories are not in excess, higher CHO diet does not promote excessive GWG.

### 4.5. Fiber Intake

In studies of the effects of nutritional patterns on glucose metabolism, fiber is a potential confounding factor because its independent effect is difficult to isolate. Due to the absorptive properties of viscous fiber, it is believed to reduce postprandial glycemia [67]. Importantly, increased fiber often presents tolerance issues since gastrointestinal disturbance is common and compliance often suffers. It is therefore not surprising that self-reported compliance was low (40–60% due to GI intolerance) in an RCT comparing a high-fiber diet (80g fiber/60% CHO/20% fat) to a lower fiber (20 g fiber/50% CHO/30% fat) diet [68]. In an early short-term crossover trial [52], fiber intolerance was described in the context of improvement in postprandial glucose (70g fibre/70% CHO vs. 31 g fiber/35% CHO), but it is possible that the improvement in glucose could have been from differences in CHO, fat, or fiber. Across the prevailing RCTs conducted in GDM [53,55,57,60,61,62,65,69,70], the fiber difference between low- and higher CHO diets was small (2–7 g), making it an unlikely explanation for improvements in glucose. Recently it was suggested by meta-analysis that fetal growth could be influenced by higher fiber intake alone; specifically, it was shown that fetal macrosomia risk was attenuated by a low-GI diet with higher fiber (vs. low-GI with lower fiber) in GDM pregnancies [71]. These findings highlight that fiber intake in the context of any dietary CHO composition may impart an influence on maternal and infant outcomes.

### 4.6. Low-Glycemic Index CHO Diets

The glycemic index of a CHO food describes its potential to raise blood glucose: A low-GI food has less potential for increasing glucose postprandially, while there is a more acute rise in blood glucose with high-GI foods [72,73]. Low-GI foods are thought to be potentially associated with reduced risk of LGA/macrosomia due to the fact that blood glucose excursions are lower with their consumption [74]. The previously mentioned meta-analysis of five RCTs [71] (n = 302 participants) further suggested that a low-GI diet (vs. higher GI) in GDM reduced the risk of macrosomia. In a highly controlled study in China [70], fasting glucose was decreased (−3.7%) in women with a diagnosis of GDM (BMI of 20–21 kg/m^2^) who were randomized to a low-GI diet for 4-days (brown-rice meals) as compared to women on a control (high-GI white rice) diet (−1.2%). Further, a greater reduction in postprandial glucose was also seen in the low-GI group (−19 to −22% vs. −7 to −12%, respectively). Total CHO and energy intake was held constant between the two groups. The preponderance of data suggest that low-GI diets may improve fasting/postprandial glucose and reduce infant birthweight in pregnancies complicated by GDM, particularly with increased fiber intake.

### 4.7. Maternal and Infant Outcomes

Two prospective RCTs of CHO intake in GDM were powered on a difference in birth weight. One RCT conducted in Australia (n = 92) [65], designed to compare the effectiveness of a low-GI diet (GI = 47) to a higher GI diet (GI = 53) on infant birth weight (260 g difference) was stopped early. This was due to an undetectable difference in birthweight—neither group met the GI goals. Women randomized to the DASH diet (vs. control) in Iran [62] had offspring with a lower birth weight. However, the difference in birth weight was uncommonly large (3223 g vs. 3819 g) for the sample size (n = 58). The intervention was of only 4 wks’ duration and dietary intake is unknown during the last 8–10 wks of pregnancy. In the pilot study by our group [57] (all food was provided), the data showed a trend for higher infant adiposity in women randomized to the lower CHO (higher fat) diet for 6–7 wks through delivery. Maternal IR at 37 wks was highly correlated with adiposity after birth (*r* = 0.731; *p* < 0.01). Thus, the limited studies prevent firm conclusions, but point to a need for future studies designed to test the effects of a restrictive vs. less-CHO-restrictive approaches to nutrition therapy on infant outcomes such as infant adiposity. Infant adiposity is a stronger predictor of childhood obesity than birthweight alone [75].

## 5. Pitfalls Associated with Nutrition Therapy in GDM

### 5.1. Anxiety and Fear as Treatment Barriers

The diagnosis of GDM, late in the second trimester, often comes as a surprise to women who had believed their pregnancy to be progressing normally until that moment [76]. Unexpectedly, the pregnancy is labeled as ‘high-risk’. Women must learn to use a glucometer, change their diet and follow a rigid CHO restriction, interpret food labels and count CHO grams, avoid habitual preferred foods, attend more doctor visits, and importantly, they are told that if they “fail” nutrition therapy, insulin injections and increased fetal surveillance will be required. Women with GDM report feelings of guilt, failure [77], and anxiety [78] with insulin therapy. With the diagnosis, women feel fear for the well-being of the baby and have higher anxiety and depression [76,78]. They have conveyed that nutrition therapy is intrusive [76], culturally unacceptable [77,78,79], and difficult to follow late in pregnancy [76,80,81]. Food selection is mentally exhausting for women with GDM, and the necessary fast adaptation to nutrition recommendations is a challenge [80]. In our population of women with GDM, the fear of macrosomia is an enormous burden, leading them to consume very low-CHO diets in which fat is instead freely consumed. They rationalize that less CHO will lead to a better growth outcome for their baby. Their glucose control may be within the target range, but in the background they have high anxiety, feel unhappy, and only consume a narrow range of “safe” foods [9,16]. For some women, the nutrition plan is a looming challenge they cannot meet, so it is not followed. This is similar to the experience described in a recent report from China [81], where women reported not following recommendations for self-monitoring of glucose because it provides feedback of their perceived failure. Thus, the socioemotional component of nutrition therapy for GDM has a dominant influence on adherence.

### 5.2. Adherence in Clinical Studies

Adherence to nutrition therapy in GDM remains a large confounding factor across clinical studies and must be considered in the interpretation of the results [9]. For example, in the RCT executed in Poland [69] (60% vs. 45% total CHO), improved glycemic control within each group was demonstrated but adherence (self-reported) to the diets was only ~50%. In Australia, investigators provided food baskets to participants to help achieve the low- vs. higher GI targets [65]. Despite this, women did not achieve the GI targets, rendering the groups too similar in GI (53 vs. 47), providing a potential explanation for the lack of difference in the outcome. In Spain [61], where insulin needs were similar in a higher vs. low-CHO diet, only 63–67% of women completed diet records, and importantly, the CHO target was met only by the low-CHO group. There was an attrition rate of 20% in the control group. Urinary sodium, a marker of adherence, was not included in the 4-week DASH studies [55,56,62]. These examples underscore the importance of considering adherence in the understanding of nutrition trial outcomes, particularly if self-report is relied upon.

## 6. Low-Carbohydrate Diets in GDM: Further Considerations

Restriction of CHO remains an available strategy for nutrition therapy in GDM and is recommended by some professional organizations, such as the American College of Obstetricians and Gynecologists [82] and the Endocrine Society [83]. Other organizations, such as the Academy of Nutrition and Dietetics in their most recent clinical guideline [10], have considered the available evidence and recognized that one nutrition strategy is unlikely to work for all women with GDM. Individualization of therapy is encouraged, which is consistent with recommendations from the American Diabetes Association guideline that applies to all persons with diabetes (not restricted to pregnancy) [84]. In light of this, several aspects important to pregnancy and GDM are relevant when considering any approach to nutrition therapy (also see Ref. [9]). First: The fetus relies on a steady supply of maternal glucose to support its growth and development, particularly in the brain [85]. Facilitated diffusion of glucose across the placenta is dependent on a higher concentration of glucose in maternal circulation vs. in fetal circulation. Extreme restriction of maternal CHO intake might challenge placental glucose transport and increases the risk for ketosis [86]. Second: the recommended daily average intake for CHO intake in pregnancy (175 g) does not account for body mass, nor does it account for *placental glucose consumption* that might be much higher than previously appreciated [87]. If a pregnant mother with obesity strictly consumes 175 g/day of CHO as part of an appropriate total energy intake for her BMI, she will consume less than the recommendation that 45–65% of total energy [43] come from CHO—an issue that may require further investigation. Third: Type and quality of CHO is an important consideration in any nutrition pattern, as not all CHOs have the same glycemic potential [72]. Last: Rigid restriction of any one macronutrient category might carry unintended consequences. In light of mounting evidence demonstrating an association between maternal TG, FFA and fetal growth [88] such as birthweight and adiposity, replacing CHO calories with fat calories should be carefully considered. If higher maternal IR increases fetal-placental exposure to glucose, lipids, and amino acids [36], then increased consumption of total and saturated fat may further exacerbate IR [89,90] and increase fetal nutrient exposure, promoting overgrowth patterns. In our randomized study of women with GDM, CHO restriction (40% of total calories, compared to 60% complex CHO) was accompanied by 20% increased postprandial FFA [16,53] and a trend for higher infant adiposity [57]. Extremes in protein intake should also be approached with caution, as both restrictive and excess intake patterns have been associated with low birth weight [24,91].

Rigid restriction of any CHO in particular may have consequences in the pre- and post-pregnancy periods as well. For example, there was an association between pre-conception CHO restriction and higher incidence of neural tube defects in a study using observational data, which is likely attributed to reduced intake of folic acid [92]. Further, it has been reported that there is blunted 24 h insulin secretion and exacerbation of IR with rigid CHO restriction (20 g/day) combined with high fat intake. The blunted insulin secretion resulted in insulin levels inadequate to suppress lipolysis and drove elevated FFA over 24 h [93]. Finally, a recent prospective observational study linked a low-CHO diet pattern (also high in protein and fat from animal sources) to increased Type 2 diabetes in women with prior GDM [94]. These data highlight the importance of consultation with a trained registered dietitian or nutritionist in tailoring nutritional intake patterns for women during the periconceptual period, and in the postpartum period after GDM has been identified.

## 7. Conclusions

Because the impact of GDM is upon both maternal and offspring metabolic health, the stakes are high to elucidate consistent, effective strategies that improve lifelong health in this important population. Some form of nutrition therapy will be required for every woman with GDM, regardless of diagnostic criteria [9]. The prevailing gestalt of the evidence demonstrates that the restriction of CHO results in improved maternal glycemia; however, similar and potentially more favorable outcomes may be achieved by less-restrictive approaches that include an optimal mixture of higher-quality CHOs with lower GI and lower fat. A less restrictive nutritional approach may ease anxiety associated with the diagnosis and plan for therapy. Low-GI diets as part of any CHO intake may reduce the need for insulin and improve post-meal glycemia. An emphasis on prescription of eucaloric diets to minimize weight gain is essential in light of data suggesting that many women with GDM do require more weight gain for fetal growth [95] beyond that already gained prior to the diagnosis.

The recent meta-analysis demonstrating that health-promoting nutritional manipulation from the usual intake pattern improves maternal glucose and lowers infant birthweight in GDM [47] creates a platform for future studies. Future RCTs should include (also described in Ref. [9]): A report of weight gained during the intervention as well as total GWG; provision of foods during intervention; measurement of infant adiposity as a marker of in-utero nutrition exposure and higher predictive value for childhood overweight (over birthweight) [75]; control of confounders such as medication, energy intake, and physical activity between diets; better biomarkers of dietary adherence; inclusion of homogeneous participants (ethnicity, glucose metabolism) [11,28]; achieved glycemia reports with the glucose targets employed [11]; and more reliable diet reporting. There is speculation that increased fetal intrahepatic lipid stores from a high simple sugar, higher fat diet that leads to excess FFA availability could potentially increase the risk of non-alcoholic fatty liver disease [96], emphasizing the need for measures of metabolic health in the offspring over birthweight or percent fat at birth. Finally, it may be that women with GDM will best respond to a nutrition plan that is customized, and the potential of personalized nutrition may be realized sooner than anticipated with advances in nutrigenomics and metabolomics. Until this is possible, the plan for nutrition therapy should include adaptations to cultural preferences, and socioemotional and economic factors so that it can be sustainable during and beyond the pregnancy for the mother and offspring.
